# Supplementary material for: Risk assessment and antibiotic prescribing decisions in children presenting to UK primary care with cough: a vignette study
Source: BMJ Open. 2020 Jul 20;10(7):e035761. doi: 10.1136/bmjopen-2019-035761 (PMC7375509; doi:10.1136/bmjopen-2019-035761)
Supplement: Supplementary data [file bmjopen-2019-035761supp001.pdf]

APPENDIX 1

Characteristics of vignettes

Vignette identifier	Vignette Text	STARWAVe risk assessment	STARWAVe recommendation
1	A 5-year-old child is brought by their parent with a 6-day history of cough. They have no history of asthma or vomiting. On examination, they are pyrexial, and have neither wheeze nor intercostal recession.	Very low (1 risk factor)	No immediate prescription
2	A 20-month-old child is brought by their parent with a 3-day history of cough. They have no history of asthma or vomiting. On examination, they are pyrexial, and have neither wheeze nor intercostal recession.	Normal (3 risk factors)	No immediate prescription
3	A 20-month-old child is brought by their parent with a 6-day history of cough. They have no history of asthma and have vomited twice in the last 24 hours. On examination, they are pyrexial, and have neither wheeze nor intercostal recession.	Normal (3 risk factors)	No immediate prescription
4	A 5-year-old child is brought by their parent with a 3-day history of cough. They have no history of asthma and have vomited twice in the last 24 hours. On examination, they are pyrexial, and have neither wheeze nor intercostal recession.	Normal (3 risk factors)	No immediate prescription
5	A 20-month-old child is brought by their parent with a 6-day history of cough. They have no history of asthma or vomiting. On examination, they are pyrexial, and have wheeze on chest auscultation. There is no intercostal recession.	Normal (3 risk factors)	No immediate prescription

6	A 5-year-old child is brought by their parent with a 3-day history of cough. They have no history of asthma or vomiting. On examination, they are pyrexial, and have wheeze on chest auscultation. There is no intercostal recession.	Normal (3 risk factors)	No immediate prescription
7	A 5-year-old child is brought by their parent with a 6-day history of cough. They have no history of asthma and have vomited twice in the last 24 hours. On examination, they are pyrexial, and have wheeze on chest auscultation. There is no intercostal recession.	Normal (3 risk factors)	No immediate prescription
8	A 20-month-old child is brought by their parent with a 3-day history of cough. They have no history of asthma and have vomited twice in the last 24 hours. On examination, they are pyrexial, and have wheeze on chest auscultation. There is no intercostal recession.	High (5 risk factors)	Consider an immediate prescription

Note: STARWAVe risk factors are underlined for salience. They were not underlined when vignettes were presented to GPs.

APPENDIX 2

Stress from Uncertainty (SfU) scale

1. I usually feel anxious when I am not sure of a diagnosis.
2. I find the uncertainty involved in patient care disconcerting.
3. Uncertainty in patient care makes me uneasy.
4. I am quite comfortable with the uncertainty in patient care.*
5. The uncertainty of patient care often troubles me.
6. When I am uncertain of a diagnosis, I imagine all sorts of bad scenarios -- patient dies, patient sues, etc.
7. I fear being held accountable for the limits of my knowledge.
8. I worry about malpractice when I do not know a patient's diagnosis.

Note: items 1-5 measure the construct “Anxiety due to Uncertainty” (Cronbach’s alpha=0.86); items 6-8 measure the construct “Concern About Bad Outcomes” (Cronbach’s alpha=0.73). *Reverse-scored item.

APPENDIX 3

Delayed prescriptions

Delayed prescriptions were administered 12% of the time (121/1008). The association between delayed prescribing and risk (as classified by GPs and by STARWAVe) is displayed below (yellow cells).

		Risk as classified by GPs			STARWAVe risk			Total
		Very low	Medium	High	Very low	Medium ("normal")	High	
Prescriptions	None	420	294	17	95	551	85	731
	Delayed	25	92	4	17	89	15	121
	Immediate	14	107	35	14	116	26	156
Total		459	493	56	126	756	126	1008

To investigate the effect of the manipulated factors on both delayed and immediate prescribing, we regressed the 3-category prescribing variable (0=no prescription, 1=delayed prescription, 2=immediate prescription) on patient age (0=5 years, 1=20 months), illness duration (0=6 days, 1=3 days), vomiting (0=absent, 1=present) and wheeze (0=absent, 1=present). This ordinal logistic regression analysis was conducted using the Stata user-written program "gologit2".[1,2] Statistical tests of the proportional odds assumption revealed that two variables met it (p_{age}=0.124 and p_{vomit}=0.522) and two did not ($p_{duration}$=0.034 and p_{wheeze}=0.003). Put differently: the respective effects of age and vomiting were consistent for successive levels of the ordinal dependent variable, while those of duration and wheeze were not. Thus, we constructed a partial proportional odds (PPO) model, where two coefficients

[1] Williams R. Generalized ordered logit/partial proportional odds models for ordinal dependent variables. *Stata J* 2006;6(1):58-82.
[2] Williams R. Understanding and interpreting generalized ordered logit models. *J Math Sociol* 2016;40:7-20.

were fixed (age and vomiting) and two were allowed to vary (duration and wheeze). A global Wald test confirmed that the proportional odds assumption was not violated in this PPO model (χ^2 (2) 2.63, p=0.268).

Results are tabulated below. The model progresses in two steps: the first step compares "no prescription" (coded 0) to "delayed prescription" and "immediate prescription" (both coded 1); the second compares "no prescription" and "delayed prescription" (both coded 0) to "immediate prescription" (coded 1). Trends were consistent across steps, and consistent with those reported in the main text. Specifically, patient age did not influence the odds of a prescription (p=0.569) and short illness duration decreased them (p<0.001). Presence of vomiting and presence of wheeze both increased prescribing odds (both *ps*<0.001). Two coefficients were allowed to vary across steps (duration and wheeze): in both cases, effects grew stronger from step 1 to step 2.

	STEP 1: no prescription (coded 0) vs. delayed/immediate (coded 1)	STEP 2: no/delayed prescription (coded 0) vs. immediate (coded 1)
Age (<2 years)	0.92 [0.69-1.23]	0.92 [0.69-1.23]
Duration (≤3 days)	0.46 [0.34-0.62]*	0.34 [0.24-0.49]*
Vomiting	1.49 [1.24-1.80]*	1.49 [1.24-1.80]*
Wheeze	2.50 [1.91-3.28]*	3.89 [2.66-5.69]*

*p<0.001. Cells contain odds ratios; square brackets contain 95% CIs. Step 2 of the model (no/delayed prescription vs. immediate) is akin to the model reported in the main text; differences in coefficients may be attributed to different estimation procedures (e.g., the ordinal model estimates all parameters simultaneously).[3]

[3] Williams R. Understanding and interpreting generalized ordered logit models. *J Math Sociol* 2016;40:7-20.

APPENDIX 4

Risk assessments and prescribing decisions per vignette

Vignette identifier	GP risk assessments			GP prescribing decisions		
	Very low	Medium	High	None	Delayed	Immediate
1	64% (81/126)	35% (44/126)	1% (1/126)	75% (95/126)	14% (17/126)	11% (14/126)
2	71% (89/126)	29% (37/126)	0% (0/126)	94% (118/126)	5% (6/126)	2% (2/126)
3	42% (52/125)	53% (66/125)	6% (7/125)	74% (93/125)	13% (16/125)	13% (16/125)
4	59% (75/127)	40% (51/127)	1% (1/127)	81% (103/127)	14% (18/127)	5% (6/127)
5	25% (32/127)	62% (79/127)	13% (16/127)	59% (75/127)	11% (14/127)	30% (38/127)
6	53% (66/125)	44% (55/125)	3% (4/125)	77% (96/125)	14% (17/125)	10% (12/125)
7	25% (31/126)	64% (80/126)	12% (15/126)	52% (66/126)	14% (18/126)	33% (42/126)
8	26% (33/126)	64% (81/126)	10% (12/126)	67% (85/126)	12% (15/126)	21% (26/126)
