# Supplementary material for: Risk assessment and antibiotic prescribing decisions in children presenting to UK primary care with cough: a vignette study
Source: BMJ Open. 2020 Jul 20;10(7):e035761. doi: 10.1136/bmjopen-2019-035761 (PMC7375509; doi:10.1136/bmjopen-2019-035761)
Supplement: Author's manuscript [file bmjopen-2019-035761.draft_revisions.pdf]

BMJ Open

BMJ Open is committed to open peer review. As part of this commitment we make the peer review history of every article we publish publicly available.

When an article is published we post the peer reviewers' comments and the authors' responses online. We also post the versions of the paper that were used during peer review. These are the versions that the peer review comments apply to.

The versions of the paper that follow are the versions that were submitted during the peer review process. They are not the versions of record or the final published versions. They should not be cited or distributed as the published version of this manuscript.

BMJ Open is an open access journal and the full, final, typeset and author-corrected version of record of the manuscript is available on our site with no access controls, subscription charges or pay-per-view fees (http://bmjopen.bmj.com).

If you have any questions on BMJ Open's open peer review process please email info.bmjopen@bmj.com

BMJ Open

Risk assessment and antibiotic prescribing decisions in children presenting with cough: a vignette study

Journal:	*BMJ Open*
Manuscript ID	bmjopen-2019-035761
Article Type:	Original research
Date Submitted by the Author:	15-Nov-2019
Complete List of Authors:	Nurek, Martine; Imperial College London, Surgery and Cancer<br>Delaney, Brendan; Imperial College London, Surgery and Cancer<br>Kostopoulou, Olga; Imperial College London, Surgery and Cancer
Keywords:	PRIMARY CARE, Respiratory infections < THORACIC MEDICINE, MEDICAL EDUCATION & TRAINING, Public health < INFECTIOUS DISEASES, Health informatics < BIOTECHNOLOGY & BIOINFORMATICS

SCHOLARONE™
Manuscripts

I, the Submitting Author has the right to grant and does grant on behalf of all authors of the Work (as defined in the below author licence), an exclusive licence and/or a non-exclusive licence for contributions from authors who are: i) UK Crown employees; ii) where BMJ has agreed a CC-BY licence shall apply, and/or iii) in accordance with the terms applicable for US Federal Government officers or employees acting as part of their official duties; on a worldwide, perpetual, irrevocable, royalty-free basis to BMJ Publishing Group Ltd ("BMJ") its licensees and where the relevant Journal is co-owned by BMJ to the co-owners of the Journal, to publish the Work in this journal and any other BMJ products and to exploit all rights, as set out in our licence.

The Submitting Author accepts and understands that any supply made under these terms is made by BMJ to the Submitting Author unless you are acting as an employee on behalf of your employer or a postgraduate student of an affiliated institution which is paying any applicable article publishing charge ("APC") for Open Access articles. Where the Submitting Author wishes to make the Work available on an Open Access basis (and intends to pay the relevant APC), the terms of reuse of such Open Access shall be governed by a Creative Commons licence – details of these licences and which Creative Commons licence will apply to this Work are set out in our licence referred to above.

Other than as permitted in any relevant BMJ Author's Self Archiving Policies, I confirm this Work has not been accepted for publication elsewhere, is not being considered for publication elsewhere and does not duplicate material already published. I confirm all authors consent to publication of this Work and authorise the granting of this licence.

For peer review only

Title:

Risk assessment and antibiotic prescribing decisions in children presenting with cough: a vignette study

Authors:

Martine Nurek*[,1], Brendan Delaney[1] and Olga Kostopoulou[1]

Author affiliations:

[1] Imperial College London, Department of Surgery and Cancer, Faculty of Medicine, 5th floor Medical School Building, St Mary's Campus, Norfolk Place, London W2 1PG, UK.

* Correspondence concerning this article should be addressed to Martine Nurek, Imperial College London, Department of Surgery and Cancer, 5th floor Medical School Building, St Mary's Campus, Norfolk Place, London W2 1PG, UK (email: m.nurek@imperial.ac.uk; phone: +44 (0)20 759 43062)

Word count:

3264

For peer review only

ABSTRACT

Objectives: The validated "STARWAVe" clinical prediction rule (CPR) uses seven variables to guide risk assessment and antimicrobial stewardship in children presenting with cough (**S**hort illness duration, **T**emperature, **A**ge, **R**ecession, **W**heeze, **A**sthma, **V**omiting). We aimed to compare General Practitioners' (GPs) risk assessments and prescribing decisions to those of STARWAVe, and assess the influence of the CPR's clinical variables.

Setting: Primary care.

Participants: 252 GPs, currently practising in the UK.

Design: GPs were randomly assigned to view four (of a possible eight) clinical vignettes online. Each vignette depicted a child presenting with cough, who was described in terms of the seven STARWAVe variables. Systematically, we manipulated patient age (20 months vs. 5 years), illness duration (3 vs. 6 days), vomiting (present vs. absent) and wheeze (present vs. absent), holding the remaining STARWAVe variables constant.

Outcome measures: Per vignette, GPs assessed risk of hospitalisation and indicated whether they would prescribe antibiotics or not.

Results: GPs overestimated risk of hospitalisation in 9% of vignette presentations (88/1008) and underestimated it in 46% (459/1008). Despite underestimating risk,

For peer review only

they overprescribed: 78% of prescriptions were unnecessary relative to GPs' own risk assessments (121/156), while 83% were unnecessary relative to STARWAVe risk assessments (130/156). All four of the manipulated variables influenced risk assessments, but only three influenced prescribing decisions: a shorter illness duration reduced prescribing odds (OR 0.14, 95% CI 0.08-0.27, p<0.001), while vomiting and wheeze increased them (OR_{vomit} 2.17, 95% CI 1.32-3.57, p=0.002; OR_{wheeze} 8.98, 95% CI 4.99-16.15, p<0.001).

Conclusions: Relative to STARWAVe, GPs underestimated risk of hospitalisation, overprescribed, and appeared to misinterpret illness duration (prescribing for longer rather than shorter illnesses). It is important to ascertain discrepancies between CPRs and current clinical practice. This has implications for the integration of CPRs into the electronic health record and the provision of intelligible explanations to decision makers.

For peer review only

STRENGTHS AND LIMITATIONS OF THIS STUDY

- This is the first study to suggest discrepancies between the STARWAVe clinical prediction rule and current clinical practice.
- Use of clinical vignettes allowed us to manipulate some variables while holding others constant; thus we could identify *causal* relationships between specific clinical variables and antibiotic prescribing decisions.
- In so doing, we bring much-needed experimental evidence to the literature, which is currently dominated by interview and observational studies.
- The disadvantage of using clinical vignettes is that our results are based on hypothetical clinical scenarios, which contained limited information.
- Moreover, we manipulated only a subset of the STARWAVe variables; future work could increase the number of clinical variables manipulated, and explore non-clinical factors too.

For peer review only

INTRODUCTION

Combatting antimicrobial resistance is high on policy agendas internationally.[1-3] One of the key means advocated is judicious antibiotic prescribing.[1] Over 80% of all NHS antibiotic prescriptions are issued in primary care,[4] where despite numerous campaigns, mandates and financial incentives, rates remain unacceptably high.[5] Despite strong evidence of only modest symptomatic benefits for acute respiratory tract infections (RTIs),[6-8] and even smaller effects against complications,[9, 10] RTIs are the most common justification for primary care antibiotic use [11] and a leading cause of overuse.[12] This is exacerbated in children, where perceived vulnerability and prognostic uncertainty (i.e., perceived risk of deterioration) lead to defensive prescribing ("treat, just in case").[12-15]

To improve risk assessment and antimicrobial prescribing in children with RTIs, a clinical prediction rule (CPR) called "STARWAVe" was recently developed and validated.[12] It was based on a large prognostic cohort study, which included 8394 children presenting to 247 general practices in England with acute cough and RTI symptoms.[12] Numerous characteristics were recorded at presentation, including demographic variables, parent-reported symptoms and physical examination signs. In a regression analysis, seven of these characteristics were found to predict hospital admission (for RTI) in the month following presentation: **S**hort illness duration ($\leq$ 3 days), **T**emperature ($\geq$ 37.8℃), **A**ge ($<$ 2 years), **R**ecession, **W**heeze, **A**sthma and **V**omiting.[12] This analysis gave rise to the "STARWAVe" clinical prediction rule: a seven-item, point-of-care checklist that can distinguish children at "very low" (0.3%, with $\leq$ 1 characteristic), "normal" (1.5%, with 2-3 characteristics) and "high" (11.8%,

For peer review only - http://bmjopen.bmj.com/site/about/guidelines.xhtml

with ≥ 4 characteristics) risk of hospitalisation, with good accuracy (area under the receiver operating characteristic curve 0.81, 95% CI 0.76-0.85).[12] Using STARWAVe, clinicians can quickly and reliably identify the “high risk” cases that might warrant antimicrobial treatment. More importantly, they can identify the “very low risk” and “normal risk” cases that will likely resolve on their own, and spare them unnecessary treatment.[12]

STARWAVe is thus a prognostic (not a diagnostic) tool. It cannot tell clinicians whether an infection is bacterial or viral. This does not however invalidate it as an antimicrobial prescribing aid, because overprescribing is so often driven by prognostic concerns.[12-15] STARWAVe recognises this and addresses it, by providing evidence-based reassurance (to clinicians and perhaps even parents) that specific children are *not* at significant risk. In so doing, it can assuage the fears and anxieties that are known to trigger unnecessary prescriptions.

Like other CPRs and clinical risk scores (e.g., QCancer), STARWAVe could be integrated into the electronic health record to guide clinicians’ risk assessments and prescribing decisions. In fact, one research group has incorporated web-based STARWAVe decision support into a multifaceted intervention that aims to improve the management of children presenting with cough in primary care (the intervention is currently undergoing clinical trial).[16] As a rule, decision support should be transparent and intelligible to the decision maker;[17] a risk score is merely a number and could be ignored, especially if it contradicts the decision maker’s intuitive assessment of risk.[18] Thus, it is important to understand whether and how GPs’

For peer review only

intuitive risk assessments and prescribing decisions differ from those of STARWAVe, and how GPs interpret the CPR's clinical variables.

To explore this, we presented GPs with clinical vignettes describing children presenting with cough. The vignettes included all seven STARWAVe variables; however, only four were manipulated (i.e., varied systematically across the vignettes). This was due to logistical constraints: these data were collected in conjunction with another study,[19] which limited the number of vignettes that we could present and thus the number of variables that we could manipulate. We chose to manipulate patient age (20 months vs. 5 years), illness duration (3 days vs. 6 days), vomiting (present vs. absent) and wheeze (present vs. absent), holding the remaining STARWAVe variables constant (temperature, asthma, recession). Fever was present in all of the vignettes, as it is a common presenting feature of childhood RTIs.[12] Asthma and recession are both associated with airflow obstruction, but wheeze (another symptom of airflow obstruction) was more common in the STARWAVe cohort;[12] therefore we chose to manipulate wheeze, and kept asthma and recession constant across vignettes (always absent). Per vignette, GPs assessed risk of hospitalisation (very low, normal or high) and indicated whether they would prescribe antibiotics or not. We compared GPs' intuitive risk assessments and prescribing decisions to STARWAVe guidelines, and assessed the influence of the manipulated STARWAVe variables.

METHOD

Participants

For peer review only

Sample size

In the STARWAVe elicitation and validation study, a young age (<2 years), a short illness duration (≤3 days), vomiting (present vs. absent) and wheeze (present vs. absent) were found to increase the odds of hospitalisation two- to three-fold (OR range 2.16-3.42; all *p*s≤0.004).[12] We powered the present study to detect effects of the same size on the decision to prescribe antibiotics. Specifically, using G*Power 3.1, we estimated that in order to detect the smallest effect (OR 2.16) in a 2-tailed logistic regression of prescribing (yes vs. no) on the four manipulated factors (with power=80% and α=0.05), 226 responses would be required.

Recruitment

By e-mail, we invited certified and practising UK GPs that had participated in previous studies by our research group. In addition, the NIHR-CRN (National Institute for Health Research Clinical Research Network) circulated our invitation e-mail to general practices across England.

Design and materials

Study materials were eight clinical vignettes that depicted children presenting to the GP with cough. Each child was described in terms of the seven STARWAVe variables. In a 2^{4-1} fractional factorial design, we manipulated patient age (20 months vs. 5 years), illness duration (3 days vs. 6 days), vomiting (present vs. absent) and

For peer review only

wheeze (present vs. absent), holding the remaining variables constant (presence of fever, absence of asthma and recession). We chose to use a fractional factorial design (rather than a full factorial design) because it delivers clear estimates of main effects, using half the number of vignettes (i.e., 8 rather than 16).[20]

Risk of hospitalisation ranged from “very low” (vignette 1 in Appendix 1) to “high” (vignette 8 in Appendix 1), but in most cases it was “normal” (vignettes 2-7 in Appendix 1). Thus, only one vignette warranted a prescription according to STARWAVe (vignette 8). Each participant was randomly assigned to view four of the eight vignettes.

Procedure

Interested participants were e-mailed a link to the study website, where they read an information sheet and provided informed consent. Thereafter, they saw 26 clinical vignettes: two pertained to this study and 24 pertained to an unrelated study conducted by our research group, concerning referral for suspected cancer.[19] The two antibiotics vignettes were presented after 33% and 66% of the cancer vignettes, respectively. The antibiotics and cancer vignettes were comparable in length and difficulty.

Twenty-four hours after completing this questionnaire, participants were e-mailed a link to a second questionnaire, which was structured in the same way; i.e., two antibiotics vignettes were evenly dispersed among 24 cancer vignettes. Importantly,

For peer review only - http://bmjopen.bmj.com/site/about/guidelines.xhtml

the four antibiotics vignettes seen by a given participant were selected at random and presented in a random order.

Following each antibiotics vignette, GPs were asked two questions:

- *In your opinion, what is the risk that this child would deteriorate, requiring hospital admission?*
    - *very low risk, e.g. 1 in 300*
    - *medium risk, e.g. 1 in 70* (in STARWAVe, this level of risk is labelled "normal")
    - *high risk, e.g. 1 in 8*
- *In your clinical judgement, what would be the best course of action?*
    - *no antibiotics prescription*
    - *antibiotics prescription*
    - *delayed antibiotics prescription*

A delayed antibiotics prescription is a forward-dated prescription, intended for use by the patient if symptoms do not improve by the specified date. Delayed prescriptions form part of the national strategy to reduce immediate prescribing.[21] They were not the focus of the present study, but were included to ensure that the options available were representative of daily practice, and that our measure of immediate prescribing was precise, i.e., not skewed by the absence of an option that is typically present.

Twenty-four hours later, participants were e-mailed a link to a third questionnaire; specifically, Gerrity et al.'s Stress from Uncertainty scale, which is one of the Physicians' Reactions to Uncertainty (PRU) scales.[22] The Stress from Uncertainty scale is a self-report measure of the extent to which physicians experience anxiety

For peer review only

due to clinical uncertainty and concern about bad outcomes.[22] We expected that GPs who experience greater Stress from Uncertainty (SfU) would also experience greater prognostic uncertainty when assessing children with RTIs, and thus be more inclined to prescribe. GPs were asked to indicate their agreement with each of the scale's eight items (presented in a random order) on a six-point Likert scale anchored at 1="strongly disagree" and 6="strongly agree" (Appendix 2).

Analyses

To investigate the effect of the manipulated factors on risk assessments and prescribing decisions, two logistic regression models were built. The first was an ordinal logistic regression model, where patient age (0=5 years, 1=20 months), illness duration (0=6 days, 1=3 days), vomiting (0=absent, 1=present) and wheeze (0=absent, 1=present) were used to predict perceived risk of hospitalisation (0=very low, 1=medium, 2=high). The second was a binary logistic regression model, where the same independent variables were used to predict prescribing decisions (0=no prescription, 1=prescription), which we dichotomised by merging "no prescription" and "delayed prescription" into a single category (national guidelines for antimicrobial prescribing treat them interchangeably [21]). For the interested reader, results pertaining to delayed prescriptions are presented in Appendix 3.

In two further logistic regression models (one ordinal and one binary), we investigated whether SfU scores (summed across items per GP) might relate to risk assessments (0=very low, 1=medium, 2=high) and prescribing decisions (0=no prescription, 1=prescription).

For peer review only

Statistical analysis was performed using Stata/MP 13.1. Specifically, the ordinal analyses were conducted using the Stata user-written program “gologit2”,[23, 24] where we computed cluster-robust standard errors to account for repeated measures (multiple responses per GP). The binary analyses were conducted using Stata’s “melogit” command,[25] where we included a random intercept for GPs.

Patient and public involvement

Patients and members of the public were not involved in the design, execution, reporting or dissemination of this research.

Ethical approval

Ethical approval for this study was obtained from the Health Research Authority (reference number 18/HRA/0021) and research sponsorship was provided by Imperial College London (JRO reference 17IC3882). All aspects of the study were conducted in the UK in 2018.

RESULTS

Descriptive statistics

We collected data from 252 GPs, with an average of 15 years’ experience in general practice post-qualification (*SD* 9.8). Half of the sample was female (52%, 131/252).

For peer review only

Eighty-six per cent were recruited via direct e-mail from the research team (217/252) and 14% via the NIHR-CRN (35/252).

Each GP saw four vignettes, yielding 1008 case presentations. GPs correctly classified risk of hospitalisation in 46% of these (461/1008; Table 1). Risk was rarely overestimated (9% of responses, 88/1008; blue cells) but frequently underestimated (46% of responses, 459/1008; green cells). Specifically, medium risk patients were classified as very low risk 46% of the time (345/756), while high risk patients were classified as very low or medium risk 90% of the time (114/126).

		**Risk as classified by GPs**			**Total**
		Very low	Medium	High	
**STARWAVe risk**	Very low	81	44	1	126
	Medium (“normal”)	345	368	43	756
	High	33	81	12	126
**Total**		459	493	56	1008

Table 1. Association between risk as classified by GPs and as classified by STARWAVe.

GPs classified risk as high only 6% of the time (56/1008) but prescribed immediately 15% of the time (156/1008), suggesting a dissociation between risk assessments and prescribing decisions. Indeed, 78% of prescriptions were not consistent with GPs’ own risk assessments (121/156; Table 2, blue cells) and 83% were not consistent with STARWAVe risk assessments (130/156; Table 2, green cells).

For peer review only

		Risk as classified by GPs			STARWAVe risk			Total
		Very low	Medium	High	Very low	Medium ("normal")	High	
Prescriptions	None/delayed	445	386	21	112	640	100	852
	Immediate	14	107	35	14	116	26	156
Total		459	493	56	126	756	126	1008

Table 2. Association between risk (as classified by GPs and by STARWAVe) and prescribing decisions.

Appendix 4 presents the number and proportion of prescriptions per vignette. The case with the highest rate of prescription was not the high risk case, which received a prescription only 21% of the time (26/126; vignette 8). Rather, it was a medium risk case, describing a 5-year-old child with a 6-day illness duration who had both vomiting and wheeze (33%, 42/126; vignette 7).

Results of planned analyses

Younger patient age (20 months vs. 5 years) increased perceived risk of hospitalisation (OR 1.49, 95% CI 1.14-1.95, p=0.003), while a short illness duration decreased it (OR 0.54, 95% CI 0.42-0.69, p<0.001). Presence of vomiting and presence of wheeze were both associated with higher risk estimates (OR_{vomit} 1.92, 95% CI 1.57-2.36, p<0.001; OR_{wheeze} 3.33, 95% CI 2.66-4.16, p<0.001). Statistical tests of the proportional odds assumption revealed that all four variables met it; i.e., the effect of each independent variable was consistent for successive levels of the ordinal dependent variable (all $ps \geq 0.099$). A global Wald test confirmed that the proportional odds assumption was not violated in this model (χ^2 (4) 4.70, p=0.320).

For peer review only

Patient age did not influence the odds of a prescription (OR 1.42, 95% CI 0.83-2.42, p=0.201), but a short illness duration decreased them (OR 0.14, 95% CI 0.08-0.27, p<0.001). Presence of vomiting and presence of wheeze both increased prescribing odds (OR_{vomit} 2.17, 95% CI 1.32-3.57, p=0.002; OR_{wheeze} 8.98, 95% CI 4.99-16.15, p<0.001). When prescribing was treated as a 3-category ordinal variable (0=no prescription, 1=delayed prescription, 2=immediate prescription), these findings did not change (Appendix 3).

SfU scores were unrelated to risk assessments (OR 1.00, 95% CI 0.98-1.02, p=0.935; proportional odds assumption met with p_{SfU}=0.406) and prescribing decisions (OR 1.00, 95% CI 0.96-1.03, p=0.875).

DISCUSSION

We compared GPs' risk assessments and antimicrobial prescribing decisions to a normative model (the STARWAVe CPR), in the context of clinical vignettes that varied the features (age, illness duration, vomiting, wheeze) of children presenting with cough. Relative to STARWAVe, GPs frequently underestimated the patient's risk of deterioration, but nonetheless overprescribed: the vast majority of their prescriptions were unnecessary relative to their own risk assessments (78%) and STARWAVe risk assessments (83%).

All four of the manipulated variables influenced GPs' risk assessments, which increased when the child was younger (20 months vs. 5 years), when illness duration was longer (6 vs. 3 days) and when vomiting and/or wheeze were present (vs.

For peer review only

absent). Comparing the odds ratios for these relationships to the STARWAVe model (Table 3), we note both similarities and discrepancies. Specifically, GPs' interpretations of patient age, vomiting and wheeze were consistent with the STARWAVe model, but their interpretation of illness duration was not: a shorter illness duration reduced – rather than increased – GP estimates of risk.

**Predictor**	**OR_{GPs}**	**$OR_{STARWAVe}$**
Age (<2 years)	1.49 [1.14-1.95]*	3.42 [2.12-5.58]*
Duration (≤3 days)	0.54 [0.42-0.69]*	2.77 [1.77-4.35]*
Vomiting	1.92 [1.57-2.36]*	2.56 [1.54-4.31]*
Wheeze	3.33 [2.66-4.16]*	2.16 [1.28-3.60]*
Temperature		1.99 [1.22-3.25]*
Asthma		3.93 [2.20-7.03]*
Recession		3.82 [2.23-6.62]*

Table 3. The effect of patient age, illness duration, vomiting and wheeze on risk of hospitalisation, according to present participants (OR_{GPs}) and STARWAVe ($OR_{STARWAVe}$). *$p \leq 0.006$. Square brackets contain 95% CIs.

Like risk perceptions, prescribing increased when illness duration was long (inverted OR 7.14) and when vomiting and/or wheeze were present (OR_{vomit} 2.17; OR_{wheeze} 8.98). Patient age had no reliable effect on prescribing (OR 1.42). Again, these findings are not entirely consistent with the STARWAVe model, but they are consistent with previous, non-experimental research. In one interview study, for example, GPs reported that they were more likely to prescribe antibiotics to children with RTIs given prolonged duration of symptoms, abnormal chest signs and (less frequently) vomiting.[13] Various observational studies have likewise identified chest

For peer review only

abnormalities [26-30] and vomiting [29] as clinical characteristics that prompt prescribing. In contrast, previous literature concerning the effect of age on prescribing is mixed: two studies found that older (vs. younger) patients were more likely to receive a prescription,[28, 31] but three identified no association between age and prescribing.[27, 29, 30]

Interestingly, the one patient that warranted a prescription received one only 21% of the time. Thus, we identified not only overprescription but underprescription too. Underprescription has been detected in previous studies; for example, one observational study (of adults presenting to their GP with cough and RTI) found that 16% of patients with a bacterial infection (pneumonia) did not receive a prescription.[29] Presently, the rate of underprescription was considerably higher (79%) and likely due to the patient's short illness duration (3 days). However, conclusions cannot be drawn on the basis of a single vignette; underprescription requires further investigation in a larger and more varied set of cases.

Risk assessments and prescribing tendencies bore no association to GPs' self-reported levels of "Stress from Uncertainty". However, Grol and colleagues found that greater willingness to take risks (as measured on their Attitudes to Risk Taking scale) was associated with significantly fewer antibiotics prescriptions for respiratory problems and URTI/common cold.[32] Attitudes toward risk – rather than attitudes toward uncertainty – may thus prove a fruitful avenue for future research.

Limitations and future work

For peer review only

The rate of prescription identified here (15%) appears lower than that observed in other studies. For example, Hay et al. identified a rate of 37% in their prospective cohort study of children presenting to the GP with cough.[12] Notably, present work included few high risk presentations (13%), but high risk presentations were likewise infrequent in the study by Hay et al. (3%).[12] The likely explanation for the lower prescription rate identified here is that our vignettes ignored the complex interpersonal dynamics of the clinical encounter, which are known to influence prescribing behaviour.[13-15, 33, 34] For example, prescription likelihood is increased by perceived pressure from patients/parents to prescribe;[14, 30, 33, 35, 36] by the desire to maintain good relationships with patients/parents;[13, 37, 38] by fear of medicolegal problems;[13, 15, 38] and by time pressure.[13, 14, 36-38] Importantly, these factors can be incorporated into clinical vignettes, as demonstrated by Sirota and colleagues; these authors found that prescriptions were twice as likely when patient pressure for antibiotics was present (vs. absent) from a clinical vignette.[33] On the one hand, it is a limitation of our vignettes that these interpersonal factors were absent; on the other, our work highlights that antibiotics are overprescribed *even when* these interpersonal factors are absent. It is worrying that so many GPs considered antibiotics to be the most appropriate course of action, not simply the most expedient one. Qualitative research may be useful to understand why GPs prescribed to patients that they deemed to be low risk, in the absence of any interpersonal pressure to do so.

Data for this study were collected in conjunction with another project, which limited the number of STARWAVe variables that we could manipulate. A comprehensive investigation of all seven STARWAVe variables would undoubtedly return new and

For peer review only

valuable insights. Future investigations might also treat the continuous STARWAVe variables (age and illness duration) as continuous (not binary), to test the generalisability of the trends identified here.

Despite these limitations, present work sheds light on the determinants of antibiotic prescribing in child RTI presentations, bringing much-needed experimental evidence to a literature that has to date relied predominantly on self-report [13-15, 31, 36, 38, 39] and observational [26-30] data. It also speaks to the difficulties that may be encountered if STARWAVe is provided as a decision aid to GPs. Firstly, GPs' classification of risk in this study was largely incompatible with STARWAVe's; GPs consistently chose lower risk than STARWAVe would suggest. Still, they prescribed more frequently than STARWAVe risk classification would support. Presenting GPs with STARWAVe's risk classification will likely exacerbate prescribing (since GPs overprescribed with their own, lower classifications of risk). Presenting them with a recommendation may also be ineffective, unless the recommendation is accompanied by an explanation. Explaining the recommendation in terms of the variables that increase/decrease a child's risk of hospitalisation may be a way forward, and enable GPs to understand why their own intuitive decision might differ from the recommendation. Identifying the factors that are likely to be misinterpreted by GPs is important when explaining the rationale behind recommendations.

COMPETING INTERESTS

Dr. Nurek, Dr. Delaney and Dr. Kostopoulou report grants from the NIHR Imperial Patient Safety Translational Research Centre, during the conduct of the study.

For peer review only

FUNDING

This work was supported by the National Institute for Health Research (NIHR) Patient Safety Translational Research Centre. The views expressed are those of the authors and not necessarily those of the NIHR or the Department of Health and Social Care. The funders had no role in the study design; in the collection, analysis and interpretation of the data; in the writing of the report; and in the decision to submit the paper for publication.

AUTHOR CONTRIBUTIONS

All authors contributed to the design of the study. MN performed the data collection; MN and OK performed the data analysis. MN drafted the manuscript; OK and BD provided critical revision.

DATA SHARING

For peer review purposes, the data are provided at https://osf.io/r3ype/?view_only=a66a7be2fcbe45a5a67a454ba5b3750a. Upon acceptance, the data will be made publicly available on the Open Science Framework under a CC-By Attribution 4.0 International Licence.

LICENSE STATEMENT

For peer review only

The Submitting Author has the right to grant and does grant on behalf of all authors of the Work (as defined in the below author licence), an exclusive licence and/or a non-exclusive licence for contributions from authors who are: i) UK Crown employees; ii) where BMJ has agreed a CC-BY licence shall apply, and/or iii) in accordance with the terms applicable for US Federal Government officers or employees acting as part of their official duties; on a worldwide, perpetual, irrevocable, royalty-free basis to BMJ Publishing Group Ltd ("BMJ") its licensees and where the relevant Journal is co-owned by BMJ to the co-owners of the Journal, to publish the Work in BMJ Open and any other BMJ products and to exploit all rights, as set out in our licence.

The Submitting Author accepts and understands that any supply made under these terms is made by BMJ to the Submitting Author unless you are acting as an employee on behalf of your employer or a postgraduate student of an affiliated institution which is paying any applicable article publishing charge ("APC") for Open Access articles. Where the Submitting Author wishes to make the Work available on an Open Access basis (and intends to pay the relevant APC), the terms of reuse of such Open Access shall be governed by a Creative Commons licence – details of these licences and which Creative Commons licence will apply to this Work are set out in our licence referred to above

REFERENCES

1. Department of Health. *UK Five Year Antimicrobial Resistance Strategy 2013 to 2018;* 2013.
2. The White House. *National Strategy for Combating Antibiotic Resistant Bacteria;* 2014. https://www.whitehouse.gov/sites/default/files/docs/carb_national_strategy.pdf. Accessed August 16, 2016.
3. World Health Organisation. *Antimicrobial Resistance: Global Report on Surveillance;* 2014. http://apps.who.int/iris/bitstream/10665/112642/1/9789241564748_eng.pdf. Accessed August 16, 2016.
4. NHS England. *The NHS Atlas of Variation in Healthcare September 2015: Reducing Unwarranted Variation to Increase Value and Improve Quality;* 2015. http://www.rightcare.nhs.uk/atlas/RC_nhsAtlas3_HIGH_150915.pdf. Accessed August 16, 2016.
5. Gulliford MC, Dregan A, Moore MV, et al. Continued high rates of antibiotic prescribing to adults with respiratory tract infection: survey of 568 UK general practices. *BMJ Open* 2014;4(10):e006245.
6. Spinks A, Glasziou PP, Del Mar CB. Antibiotics for sore throat. *Cochrane Database Syst Rev* 2013(11).
7. Smith SM, Fahey T, Smucny J, et al. Antibiotics for acute bronchitis*. Cochrane Database Syst Rev*, 2014(3).
8. Venekamp RP, Sanders SL, Glasziou PP, et al. Antibiotics for acute otitis media in children. *Cochrane Database Syst Rev* 2015(6).

For peer review only

9. Petersen I, Johnson AM, Islam, A, et al. Protective effect of antibiotics against serious complications of common respiratory tract infections: retrospective cohort study with the UK General Practice Research Database. *BMJ* 2007;335(7627):982.

10. Fahey T, Stocks N, Thomas T. Systematic review of the treatment of upper respiratory tract infection. *Arch Dis Child* 1998;79(3):225-230.

11. McCormick A, Fleming D, Charlton J, Office of Population Censuses and Surveys, Department of Health, Royal College of General Practitioners. *Morbidity Statistics from General Practice. Fourth National Study 1991-1992.* London: H.M.S.O.

12. Hay AD, Redmond NM, Turnbull, S, et al. Development and internal validation of a clinical rule to improve antibiotic use in children presenting to primary care with acute respiratory tract infection and cough: a prognostic cohort study. *Lancet Respir Med* 2016;4(11):902-910.

13. Horwood J, Cabral C, Hay AD, et al. Primary care clinician antibiotic prescribing decisions in consultations for children with RTIs: a qualitative interview study. *Br J Gen Pract* 2016;66(644):e207-e213.

14. Lucas PJ, Cabral C, Hay AD, et al. A systematic review of parent and clinician views and perceptions that influence prescribing decisions in relation to acute childhood infections in primary care. *Scand J Prim Health Care* 2015;33(1):11-20.

15. Cabral C, Lucas PJ, Ingram J, et al. “It's safer to…” parent consulting and clinician antibiotic prescribing decisions for children with respiratory tract infections: an analysis across four qualitative studies. *Soc Sci Med* 2015;136:156-164.

For peer review only

16. Turnbull SL, Redmond NM, Lucas P, et al. The CHICO (Children's Cough) Trial protocol: a feasibility randomised controlled trial investigating the clinical and cost-effectiveness of a complex intervention to improve the management of children presenting to primary care with acute respiratory tract infection. *BMJ Open* 2015;5(9):e008615.

17. Doshi-Velez, F, Kim B. Towards a rigorous science of interpretable machine learning. arXiv:1702.08608, 2017.

18. Chiang PP-C, Glance D, Walker J, et al. Implementing a QCancer risk tool into general practice consultations: an exploratory study using simulated consultations with Australian general practitioners. *Br J Cancer* 2015;112(s1):S77.

19. Kostopoulou O, Nurek M, Delaney BC. Disentangling the relationship between physician and organizational performance: a signal detection approach. Manuscript under review.

20. Wu CFJ, Hamada MS. Experiments: Planning, Analysis, and Optimization. Hoboken, New Jersey: John Wiley & Sons 2009.

21. Guidance from the National Institute for Health and Care Excellence. Respiratory tract infections – antibiotic prescribing: prescribing of antibiotics for self-limiting respiratory tract infections in adults and children in primary care. http://www.nice.org.uk/guidance/cg69 (accessed 23 May 2019).

22. Gerrity MS, White KP, DeVellis, RF, et al. Physicians' Reactions to Uncertainty: refining the constructs and scales. *Motiv Emot* 1995;19(3):175-191.

23. Williams R. Understanding and interpreting generalized ordered logit models. *J Math Sociol* 2016;40(1):7-20.

For peer review only

24. Williams R. Generalized ordered logit/partial proportional odds models for ordinal dependent variables. *Stata J* 2006;6(1):58-82.

25. StataCorp. Stata multilevel mixed-effects reference manual: release 13. College Station, TX;2013.

26. Fischer T, Fischer S, Kochen, MM, et al. Influence of patient symptoms and physical findings on general practitioners' treatment of respiratory tract infections: a direct observation study. *BMC Fam Pract* 2005;6(1):6.

27. Holmes WF, Macfarlane JT, Macfarlane RM, et al. Symptoms, signs, and prescribing for acute lower respiratory tract illness. *Br J Gen Pract* 2001;51(464):177-181.

28. Macfarlane J, Lewis SA, Macfarlane R, et al. Contemporary use of antibiotics in 1089 adults presenting with acute lower respiratory tract illness in general practice in the UK: implications for developing management guidelines. *Respir Med* 1997;91(7):427-434.

29. Hopstaken RM, Butler CC, Muris JWM, et al. Do clinical findings in lower respiratory tract infection help general practitioners prescribe antibiotics appropriately? An observational cohort study in general practice. *Fam Pract* 2005;23(2):180-187.

30. Jakobsen KA, Melbye H, Kelly MJ, et al. Influence of CRP testing and clinical findings on antibiotic prescribing in adults presenting with acute cough in primary care. *Scand J Prim Health Care* 2010;28(4):229-236.

31. Brookes-Howell L, Hood K, Cooper L, et al. Clinical influences on antibiotic prescribing decisions for lower respiratory tract infection: a nine country qualitative study of variation in care. *BMJ Open* 2012;2(3):e000795.

For peer review only

32. Grol R, Whitfield M, De Maeseneer J, et al. Attitudes to risk taking in medical decision making among British, Dutch and Belgian general practitioners. *Br J Gen Pract* 1990;40(333):134-136.

33. Sirota M, Round T, Samaranayaka S, et al. Expectations for antibiotics increase their prescribing: causal evidence about localized impact. *Health Psychology* 2017;36(4):402.

34. Howie JG. Clinical judgement and antibiotic use in general practice. *Br Med J*, 1976;2(6043):1061-1064.

35. Mangione-Smith R, McGlynn EA, Elliott MN, et al. The relationship between perceived parental expectations and pediatrician antimicrobial prescribing behavior. *Pediatrics* 1999;103(4):711-718.

36. Dempsey PP, Businger AC, Whaley LE, et al. Primary care clinicians' perceptions about antibiotic prescribing for acute bronchitis: a qualitative study. *BMC Fam Pract* 2014;15(1).

37. Petursson P. GPs' reasons for "non-pharmacological" prescribing of antibiotics: a phenomenological study. *Scand J Prim Health Care* 2005;23(2):120-125.

38. Butler CC, Rollnick S, Pill R, et al. Understanding the culture of prescribing: qualitative study of general practitioners' and patients' perceptions of antibiotics for sore throats. *BMJ* 1998;317(7159):637-642.

39. Kumar S, Little P, Britten N. Why do general practitioners prescribe antibiotics for sore throat? Grounded theory interview study. *BMJ* 2003;326(7381):138.

For peer review only

APPENDIX 1

Characteristics of vignettes

Vignette identifier	Vignette Text	STARWAVe risk assessment	STARWAVe recommendation
1	A 5-year-old child is brought by their parent with a 6-day history of cough. They have no history of asthma or vomiting. On examination, they are pyrexial, and have neither wheeze nor intercostal recession.	Very low (1 risk factor)	No immediate prescription
2	A 20-month-old child is brought by their parent with a 3-day history of cough. They have no history of asthma or vomiting. On examination, they are pyrexial, and have neither wheeze nor intercostal recession.	Normal (3 risk factors)	No immediate prescription
3	A 20-month-old child is brought by their parent with a 6-day history of cough. They have no history of asthma and have vomited twice in the last 24 hours. On examination, they are pyrexial, and have neither wheeze nor intercostal recession.	Normal (3 risk factors)	No immediate prescription
4	A 5-year-old child is brought by their parent with a 3-day history of cough. They have no history of asthma and have vomited twice in the last 24 hours. On examination, they are pyrexial, and have neither wheeze nor intercostal recession.	Normal (3 risk factors)	No immediate prescription
5	A 20-month-old child is brought by their parent with a 6-day history of cough. They have no history of asthma or vomiting. On examination, they are pyrexial, and have wheeze on chest auscultation. There is no intercostal recession.	Normal (3 risk factors)	No immediate prescription

For peer review only

6	A 5-year-old child is brought by their parent with a 3-day history of cough. They have no history of asthma or vomiting. On examination, they are pyrexial, and have wheeze on chest auscultation. There is no intercostal recession.	Normal (3 risk factors)	No immediate prescription
7	A 5-year-old child is brought by their parent with a 6-day history of cough. They have no history of asthma and have vomited twice in the last 24 hours. On examination, they are pyrexial, and have wheeze on chest auscultation. There is no intercostal recession.	Normal (3 risk factors)	No immediate prescription
8	A 20-month-old child is brought by their parent with a 3-day history of cough. They have no history of asthma and have vomited twice in the last 24 hours. On examination, they are pyrexial, and have wheeze on chest auscultation. There is no intercostal recession.	High (5 risk factors)	Consider an immediate prescription

Note: STARWAVe risk factors are underlined for salience. They were not underlined when vignettes were presented to GPs.

For peer review only

APPENDIX 2

Stress from Uncertainty (SfU) scale

1. I usually feel anxious when I am not sure of a diagnosis.
2. I find the uncertainty involved in patient care disconcerting.
3. Uncertainty in patient care makes me uneasy.
4. I am quite comfortable with the uncertainty in patient care.*
5. The uncertainty of patient care often troubles me.
6. When I am uncertain of a diagnosis, I imagine all sorts of bad scenarios -- patient dies, patient sues, etc.
7. I fear being held accountable for the limits of my knowledge.
8. I worry about malpractice when I do not know a patient's diagnosis.

Note: items 1-5 measure the construct “Anxiety due to Uncertainty” (Cronbach’s alpha=0.86); items 6-8 measure the construct “Concern About Bad Outcomes” (Cronbach’s alpha=0.73). *Reverse-scored item.

For peer review only

APPENDIX 3

Delayed prescriptions

Delayed prescriptions were administered 12% of the time (121/1008). The association between delayed prescribing and risk (as classified by GPs and by STARWAVe) is displayed below (yellow cells).

		Risk as classified by GPs			STARWAVe risk			Total
		Very low	Medium	High	Very low	Medium ("normal")	High	
Prescriptions	None	420	294	17	95	551	85	731
	Delayed	25	92	4	17	89	15	121
	Immediate	14	107	35	14	116	26	156
**Total**		459	493	56	126	756	126	1008

To investigate the effect of the manipulated factors on both delayed and immediate prescribing, we regressed the 3-category prescribing variable (0=no prescription, 1=delayed prescription, 2=immediate prescription) on patient age (0=5 years, 1=20 months), illness duration (0=6 days, 1=3 days), vomiting (0=absent, 1=present) and wheeze (0=absent, 1=present). This ordinal logistic regression analysis was conducted using the Stata user-written program "gologit2".[23, 24] Statistical tests of the proportional odds assumption revealed that two variables met it (p_{age}=0.124 and p_{vomit}=0.522) and two did not ($p_{duration}$=0.034 and p_{wheeze}=0.003). Put differently: the respective effects of age and vomiting were consistent for successive levels of the ordinal dependent variable, while those of duration and wheeze were not. Thus, we constructed a partial proportional odds (PPO) model, where two coefficients were fixed (age and vomiting) and two were allowed to vary (duration and wheeze). A global Wald test confirmed that the proportional odds assumption was not violated in this PPO model (χ^2 (2) 2.63, p=0.268).

For peer review only

Results are tabulated below. The model progresses in two steps: the first step compares “no prescription” (coded 0) to “delayed prescription” and “immediate prescription” (both coded 1); the second compares “no prescription” and “delayed prescription” (both coded 0) to “immediate prescription” (coded 1). Trends were consistent across steps, and consistent with those reported in the main text. Specifically, patient age did not influence the odds of a prescription (*p*=0.569) and short illness duration decreased them (*p*<0.001). Presence of vomiting and presence of wheeze both increased prescribing odds (both *ps*<0.001). Two coefficients were allowed to vary across steps (duration and wheeze): in both cases, effects grew stronger from step 1 to step 2.

	STEP 1:<br>no prescription (coded 0) vs. delayed/immediate (coded 1)	STEP 2:<br>no/delayed prescription (coded 0) vs. immediate (coded 1)
Age (<2 years)	0.92 [0.69-1.23]	0.92 [0.69-1.23]
Duration (≤3 days)	0.46 [0.34-0.62]*	0.34 [0.24-0.49]*
Vomiting	1.49 [1.24-1.80]*	1.49 [1.24-1.80]*
Wheeze	2.50 [1.91-3.28]*	3.89 [2.66-5.69]*

**p*<0.001. Cells contain odds ratios; square brackets contain 95% CIs. Step 2 of the model (no/delayed prescription vs. immediate) is akin to the model reported in the main text; differences in coefficients may be attributed to different estimation procedures (e.g., the ordinal model estimates all parameters simultaneously).[23]

APPENDIX 4

Risk assessments and prescribing decisions per vignette

Vignette identifier	GP risk assessments			GP prescribing decisions		
	Very low	Medium	High	None	Delayed	Immediate
1	64% (81/126)	35% (44/126)	1% (1/126)	75% (95/126)	14% (17/126)	11% (14/126)
2	71% (89/126)	29% (37/126)	0% (0/126)	94% (118/126)	5% (6/126)	2% (2/126)
3	42% (52/125)	53% (66/125)	6% (7/125)	74% (93/125)	13% (16/125)	13% (16/125)
4	59% (75/127)	40% (51/127)	1% (1/127)	81% (103/127)	14% (18/127)	5% (6/127)
5	25% (32/127)	62% (79/127)	13% (16/127)	59% (75/127)	11% (14/127)	30% (38/127)
6	53% (66/125)	44% (55/125)	3% (4/125)	77% (96/125)	14% (17/125)	10% (12/125)
7	25% (31/126)	64% (80/126)	12% (15/126)	52% (66/126)	14% (18/126)	33% (42/126)
8	26% (33/126)	64% (81/126)	10% (12/126)	67% (85/126)	12% (15/126)	21% (26/126)

For peer review only

BMJ Open

Risk assessment and antibiotic prescribing decisions in children presenting to UK primary care with cough: a vignette study

Journal:	*BMJ Open*
Manuscript ID	bmjopen-2019-035761.R1
Article Type:	Original research
Date Submitted by the Author:	09-Mar-2020
Complete List of Authors:	Nurek, Martine; Imperial College London, Surgery and Cancer<br>Delaney, Brendan; Imperial College London, Surgery and Cancer<br>Kostopoulou, Olga; Imperial College London, Surgery and Cancer
**Primary Subject Heading**:	General practice / Family practice
Secondary Subject Heading:	Public health, Medical education and training, Health informatics, Infectious diseases, Respiratory medicine
Keywords:	PRIMARY CARE, Respiratory infections < THORACIC MEDICINE, MEDICAL EDUCATION & TRAINING, Public health < INFECTIOUS DISEASES, Health informatics < BIOTECHNOLOGY & BIOINFORMATICS

SCHOLARONE™
Manuscripts

I, the Submitting Author has the right to grant and does grant on behalf of all authors of the Work (as defined in the below author licence), an exclusive licence and/or a non-exclusive licence for contributions from authors who are: i) UK Crown employees; ii) where BMJ has agreed a CC-BY licence shall apply, and/or iii) in accordance with the terms applicable for US Federal Government officers or employees acting as part of their official duties; on a worldwide, perpetual, irrevocable, royalty-free basis to BMJ Publishing Group Ltd ("BMJ") its licensees and where the relevant Journal is co-owned by BMJ to the co-owners of the Journal, to publish the Work in this journal and any other BMJ products and to exploit all rights, as set out in our licence.

The Submitting Author accepts and understands that any supply made under these terms is made by BMJ to the Submitting Author unless you are acting as an employee on behalf of your employer or a postgraduate student of an affiliated institution which is paying any applicable article publishing charge ("APC") for Open Access articles. Where the Submitting Author wishes to make the Work available on an Open Access basis (and intends to pay the relevant APC), the terms of reuse of such Open Access shall be governed by a Creative Commons licence – details of these licences and which Creative Commons licence will apply to this Work are set out in our licence referred to above.

Other than as permitted in any relevant BMJ Author's Self Archiving Policies, I confirm this Work has not been accepted for publication elsewhere, is not being considered for publication elsewhere and does not duplicate material already published. I confirm all authors consent to publication of this Work and authorise the granting of this licence.

For peer review only

Title:

Risk assessment and antibiotic prescribing decisions in children presenting to UK primary care with cough: a vignette study

Authors:

Martine Nurek*,[1], Brendan Delaney[1] and Olga Kostopoulou[1]

Author affiliations:

[1] Imperial College London, Department of Surgery and Cancer, Faculty of Medicine, 5th floor Medical School Building, St Mary's Campus, Norfolk Place, London W2 1PG, UK.

* Correspondence concerning this article should be addressed to Martine Nurek, Imperial College London, Department of Surgery and Cancer, 5th floor Medical School Building, St Mary's Campus, Norfolk Place, London W2 1PG, UK (email: m.nurek@imperial.ac.uk; phone: +44 (0)20 759 43062)

Word count:

4000

For peer review only

ABSTRACT

Objectives: The validated “STARWAVe” clinical prediction rule (CPR) uses seven variables to guide risk assessment and antimicrobial stewardship in children presenting with cough (**S**hort illness duration, **T**emperature, **A**ge, **R**ecession, **W**heeze, **A**sthma, **V**omiting). We aimed to compare General Practitioners’ (GPs) risk assessments and prescribing decisions to those of STARWAVe, and assess the influence of the CPR’s clinical variables.

Setting: Primary care.

Participants: 252 GPs, currently practising in the UK.

Design: GPs were randomly assigned to view four (of a possible eight) clinical vignettes online. Each vignette depicted a child presenting with cough, who was described in terms of the seven STARWAVe variables. Systematically, we manipulated patient age (20 months vs. 5 years), illness duration (3 vs. 6 days), vomiting (present vs. absent) and wheeze (present vs. absent), holding the remaining STARWAVe variables constant.

Outcome measures: Per vignette, GPs assessed risk of hospitalisation and indicated whether they would prescribe antibiotics or not.

Results: GPs overestimated risk of hospitalisation in 9% of vignette presentations (88/1008) and underestimated it in 46% (459/1008). Despite underestimating risk, they

For peer review only

For peer review only - http://bmjopen.bmj.com/site/about/guidelines.xhtml

overprescribed: 78% of prescriptions were unnecessary relative to GPs' own risk assessments (121/156), while 83% were unnecessary relative to STARWAVe risk assessments (130/156). All four of the manipulated variables influenced risk assessments, but only three influenced prescribing decisions: a shorter illness duration reduced prescribing odds (OR 0.14, 95% CI 0.08-0.27, $p<0.001$), while vomiting and wheeze increased them (OR_{vomit} 2.17, 95% CI 1.32-3.57, $p=0.002$; OR_{wheeze} 8.98, 95% CI 4.99-16.15, $p<0.001$).

Conclusions: Relative to STARWAVe, GPs underestimated risk of hospitalisation, overprescribed, and appeared to misinterpret illness duration (prescribing for longer rather than shorter illnesses). It is important to ascertain discrepancies between CPRs and current clinical practice. This has implications for the integration of CPRs into the electronic health record and the provision of intelligible explanations to decision makers.

For peer review only

STRENGTHS AND LIMITATIONS OF THIS STUDY

- This is the first study to suggest discrepancies between the STARWAVe clinical prediction rule and current clinical practice.
- Use of clinical vignettes allowed us to manipulate some variables while holding others constant; thus we could identify *causal* relationships between specific clinical variables and antibiotic prescribing decisions.
- In so doing, we bring much-needed experimental evidence to the literature, which is currently dominated by interview and observational studies.
- The disadvantage of using clinical vignettes is that our results are based on hypothetical clinical scenarios, which contained limited information.
- Moreover, we manipulated only a subset of the STARWAVe variables; future work could increase the number of clinical variables manipulated, and explore non-clinical factors too.

For peer review only

INTRODUCTION

Combatting antimicrobial resistance is high on policy agendas internationally.[1-3] One of the key means advocated is judicious antibiotic prescribing.[1] Over 80% of all NHS antibiotic prescriptions are issued in primary care,[4] where despite numerous campaigns, mandates and financial incentives, rates remain unacceptably high.[5] Despite strong evidence of only modest symptomatic benefits for acute respiratory tract infections (RTIs),[6-8] and even smaller effects against complications,[9, 10] RTIs are the most common justification for primary care antibiotic use [11] and a leading cause of overuse.[12] This is exacerbated in children, where perceived vulnerability and prognostic uncertainty (i.e., perceived risk of deterioration) lead to defensive prescribing ("treat, just in case").[12-15]

To improve risk assessment and antimicrobial prescribing in children with RTIs, a clinical prediction rule (CPR) called "STARWAVe" was recently developed and validated.[12] It was based on a large prognostic cohort study, which included 8394 children presenting to 247 general practices in England with acute cough and RTI symptoms.[12] Numerous characteristics were recorded at presentation, including demographic variables, parent-reported symptoms and physical examination signs. In a regression analysis, seven of these characteristics were found to predict hospital admission (for RTI) in the month following presentation: **S**hort illness duration (≤ 3 days), **T**emperature (≥ 37.8℃), **A**ge (< 2 years), **R**ecession, **W**heeze, **A**sthma and **V**omiting.[12] This analysis gave rise to the "STARWAVe" clinical prediction rule: a seven-item, point-of-care checklist that can distinguish children at "very low" (0.3%, with ≤ 1 characteristic), "normal" (1.5%, with 2-3 characteristics) and "high" (11.8%,

For peer review only - http://bmjopen.bmj.com/site/about/guidelines.xhtml

with ≥4 characteristics) risk of hospitalisation, with good accuracy (area under the receiver operating characteristic curve 0.81, 95% CI 0.76-0.85).[12] Using STARWAVe, clinicians can quickly and reliably identify the “high risk” cases that might warrant antimicrobial treatment. More importantly, they can identify the “very low risk” and “normal risk” cases that will likely resolve on their own, and spare them unnecessary treatment.[12]

STARWAVe is thus a prognostic (not a diagnostic) tool. It cannot tell clinicians whether an infection is bacterial or viral. This does not however invalidate it as an antimicrobial prescribing aid, because overprescribing is so often driven by prognostic concerns.[12-15] STARWAVe recognises this and addresses it, by providing evidence-based reassurance (to clinicians and perhaps even parents) that specific children are *not* at significant risk. In so doing, it can assuage the fears and anxieties that are known to trigger unnecessary prescriptions.

Like other CPRs and clinical risk scores (e.g., QCancer), STARWAVe could be integrated into the electronic health record to guide clinicians’ risk assessments and prescribing decisions. In fact, one research group has incorporated web-based STARWAVe decision support into a multifaceted intervention that aims to improve the management of children presenting with cough in primary care (the intervention is currently undergoing clinical trial).[16] As a rule, decision support should be transparent and intelligible to the decision maker;[17] a risk score is merely a number and could be ignored, especially if it contradicts the decision maker’s intuitive assessment of risk.[18] Thus, it is important to understand whether and how GPs’

For peer review only - http://bmjopen.bmj.com/site/about/guidelines.xhtml

intuitive risk assessments and prescribing decisions differ from those of STARWAVe, and how GPs interpret the CPR's clinical variables.

To explore this, we presented GPs with clinical vignettes describing children presenting with cough. The vignettes included all seven STARWAVe variables; however, only four were manipulated (i.e., varied systematically across the vignettes). This was due to logistical constraints: these data were collected in conjunction with another study, which limited the number of vignettes that we could present and thus the number of variables that we could manipulate. We chose to manipulate patient age (20 months vs. 5 years), illness duration (3 days vs. 6 days), vomiting (present vs. absent) and wheeze (present vs. absent), holding the remaining STARWAVe variables constant (temperature, asthma, recession). Fever was present in all of the vignettes, as it is a common presenting feature of childhood RTIs.[12] Asthma and recession are both associated with airflow obstruction, but wheeze (another symptom of airflow obstruction) was more common in the STARWAVe cohort;[12] therefore we chose to manipulate wheeze, and kept asthma and recession constant across vignettes (always absent). Per vignette, GPs assessed risk of hospitalisation (very low, normal or high) and indicated whether they would prescribe antibiotics or not. We compared GPs' intuitive risk assessments and prescribing decisions to STARWAVe guidelines, and assessed the influence of the manipulated STARWAVe variables.

METHOD

Participants

For peer review only - http://bmjopen.bmj.com/site/about/guidelines.xhtml

Sample size

In the STARWAVe elicitation and validation study, a young age (<2 years), a short illness duration (≤3 days), vomiting (present vs. absent) and wheeze (present vs. absent) were found to increase the odds of hospitalisation two- to three-fold (OR range 2.16-3.42; all *p*s≤0.004).[12] We powered the present study to detect effects of the same size on the decision to prescribe antibiotics. Specifically, using G*Power 3.1, we estimated that in order to detect the smallest effect (OR 2.16) in a 2-tailed logistic regression of prescribing (yes vs. no) on the four manipulated factors (with power=80% and α=0.05), 226 responses would be required.

Recruitment

By e-mail, we invited certified and practising UK GPs that had participated in previous studies by our research group. In addition, the NIHR-CRN (National Institute for Health Research Clinical Research Network) circulated our invitation e-mail to general practices across England.

Design and materials

Study materials were eight clinical vignettes that depicted children presenting to the GP with cough. Each child was described in terms of the seven STARWAVe variables. In a 2^{4-1} fractional factorial design, we manipulated patient age (20 months vs. 5 years), illness duration (3 days vs. 6 days), vomiting (present vs. absent) and wheeze (present vs. absent), holding the remaining variables constant (presence of fever,

For peer review only - http://bmjopen.bmj.com/site/about/guidelines.xhtml
For peer review only

absence of asthma and recession). We chose to use a fractional factorial design (rather than a full factorial design) because it delivers clear estimates of main effects, using half the number of vignettes (i.e., 8 rather than 16).[19]

Risk of hospitalisation ranged from “very low” (vignette 1 in Appendix 1) to “high” (vignette 8 in Appendix 1), but in most cases it was “normal” (vignettes 2-7 in Appendix 1). Thus, only one vignette warranted a prescription according to STARWAVe (vignette 8). Each participant was randomly assigned to view four of the eight vignettes.

Procedure

Interested participants were e-mailed a link to the study website, where they read an information sheet and provided informed consent. Thereafter, they saw 26 clinical vignettes: two pertained to this study and 24 pertained to an unrelated study conducted by our research group, concerning referral for suspected cancer. The two antibiotics vignettes were presented after 33% and 66% of the cancer vignettes respectively, and were introduced as follows: *“We understand that this is somewhat monotonous, so here is something quite different to help you re-engage attention”*. The antibiotics and cancer vignettes were comparable in length and difficulty.

Twenty-four hours after completing this questionnaire, participants were e-mailed a link to a second questionnaire, which was structured in the same way; i.e., two antibiotics vignettes were evenly dispersed among 24 cancer vignettes. Importantly,

For peer review only - http://bmjopen.bmj.com/site/about/guidelines.xhtml

the four antibiotics vignettes seen by a given participant were selected at random and presented in a random order.

Following each antibiotics vignette, GPs were asked two questions:

- *In your opinion, what is the risk that this child would deteriorate, requiring hospital admission?*
    - *very low risk, e.g. 1 in 300*
    - *medium risk, e.g. 1 in 70* (in STARWAVe, this level of risk is labelled “normal”)
    - *high risk, e.g. 1 in 8*
- *In your clinical judgement, what would be the best course of action?*
    - *no antibiotics prescription*
    - *antibiotics prescription*
    - *delayed antibiotics prescription*

A delayed antibiotics prescription is a forward-dated prescription, intended for use by the patient if symptoms do not improve by the specified date. Delayed prescriptions form part of the national strategy to reduce immediate prescribing.[20] They were not the focus of the present study, but were included to ensure that the options available were representative of daily practice, and that our measure of immediate prescribing was precise, i.e., not skewed by the absence of an option that is typically present.

Twenty-four hours later, participants were e-mailed a link to a third questionnaire; specifically, Gerrity et al.’s Stress from Uncertainty scale, which is one of the Physicians’ Reactions to Uncertainty (PRU) scales.[21] The Stress from Uncertainty scale is a self-report measure of the extent to which physicians experience anxiety

For peer review only - http://bmjopen.bmj.com/site/about/guidelines.xhtml

due to clinical uncertainty and concern about bad outcomes.[21] We expected that GPs who experience greater Stress from Uncertainty (SfU) would also experience greater prognostic uncertainty when assessing children with RTIs, and thus be more inclined to prescribe. GPs were asked to indicate their agreement with each of the scale's eight items (presented in a random order) on a six-point Likert scale anchored at 1="strongly disagree" and 6="strongly agree" (Appendix 2).

Analyses

To investigate the effect of the manipulated factors on risk assessments and prescribing decisions, two logistic regression models were built. The first was an ordinal logistic regression model, where patient age (0=5 years, 1=20 months), illness duration (0=6 days, 1=3 days), vomiting (0=absent, 1=present) and wheeze (0=absent, 1=present) were used to predict perceived risk of hospitalisation (0=very low, 1=medium, 2=high). The second was a binary logistic regression model, where the same independent variables were used to predict prescribing decisions (0=no prescription, 1=prescription), which we dichotomised by merging "no prescription" and "delayed prescription" into a single category (national guidelines for antimicrobial prescribing treat them interchangeably [20]). For the interested reader, results pertaining to delayed prescriptions are presented in Appendix 3.

In two further logistic regression models (one ordinal and one binary), we investigated whether SfU scores (summed across items per GP) might relate to risk assessments (0=very low, 1=medium, 2=high) and prescribing decisions (0=no prescription, 1=prescription).

For peer review only - http://bmjopen.bmj.com/site/about/guidelines.xhtml

Statistical analysis was performed using Stata/MP 13.1. Specifically, the ordinal analyses were conducted using the Stata user-written program "gologit2",[22, 23] where we computed cluster-robust standard errors to account for repeated measures (multiple responses per GP). The binary analyses were conducted using Stata's "melogit" command,[24] where we included a random intercept for GPs.

Patient and public involvement

Patients and members of the public were not involved in the design, execution, reporting or dissemination of this research.

Ethical approval

Ethical approval for this study was obtained from the Health Research Authority (reference number 18/HRA/0021) and research sponsorship was provided by Imperial College London (JRO reference 17IC3882). All aspects of the study were conducted in the UK in 2018.

RESULTS

Descriptive statistics

We collected data from 254 GPs. Of these, two gave only partial data and thus were excluded from the analyses. The final sample comprised 252 GPs, with an average of

For peer review only

15 years' experience in general practice post-qualification (*SD* 9.8). Half of the sample was female (52%, 131/252). Eighty-six per cent were recruited via direct e-mail from the research team (217/252) and 14% via the NIHR-CRN (35/252).

Each GP saw four vignettes, yielding 1008 case presentations. GPs correctly classified risk of hospitalisation in 46% of these (461/1008; Table 1). Risk was rarely overestimated (9% of responses, 88/1008; blue cells) but frequently underestimated (46% of responses, 459/1008; green cells). Specifically, medium risk patients were classified as very low risk 46% of the time (345/756), while high risk patients were classified as very low or medium risk 90% of the time (114/126).

		**Risk as classified by GPs**			**Total**
		Very low	Medium	High	
**STARWAVe risk**	Very low	81	44	1	126
	Medium ("normal")	345	368	43	756
	High	33	81	12	126
**Total**		459	493	56	1008

Table 1. Association between risk as classified by GPs and as classified by STARWAVe.

GPs classified risk as high only 6% of the time (56/1008) but prescribed immediately 15% of the time (156/1008), suggesting a dissociation between risk assessments and prescribing decisions. Indeed, 78% of prescriptions were not consistent with GPs' own risk assessments (121/156; Table 2, blue cells) and 83% were not consistent with STARWAVe risk assessments (130/156; Table 2, green cells).

For peer review only - http://bmjopen.bmj.com/site/about/guidelines.xhtml

		Risk as classified by GPs			STARWAVe risk			Total
		Very low	Medium	High	Very low	Medium ("normal")	High	
Prescriptions	None/delayed	445	386	21	112	640	100	852
	Immediate	14	107	35	14	116	26	156
Total		459	493	56	126	756	126	1008

Table 2. Association between risk (as classified by GPs and by STARWAVe) and prescribing decisions.

Appendix 4 presents the number and proportion of prescriptions per vignette. The case with the highest rate of prescription was not the high risk case, which received a prescription only 21% of the time (26/126; vignette 8). Rather, it was a medium risk case, describing a 5-year-old child with a 6-day illness duration who had both vomiting and wheeze (33%, 42/126; vignette 7).

Results of planned analyses

Younger patient age (20 months vs. 5 years) increased perceived risk of hospitalisation (OR 1.49, 95% CI 1.14-1.95, p=0.003), while a short illness duration decreased it (OR 0.54, 95% CI 0.42-0.69, p<0.001). Presence of vomiting and presence of wheeze were both associated with higher risk estimates (OR_{vomit} 1.92, 95% CI 1.57-2.36, p<0.001; OR_{wheeze} 3.33, 95% CI 2.66-4.16, p<0.001). Statistical tests of the proportional odds assumption revealed that all four variables met it; i.e., the effect of each independent variable was consistent for successive levels of the ordinal dependent variable (all $ps \geq 0.099$). A global Wald test confirmed that the proportional odds assumption was not violated in this model (χ^2 (4) 4.70, p=0.320).

For peer review only - http://bmjopen.bmj.com/site/about/guidelines.xhtml

Patient age did not influence the odds of a prescription (OR 1.42, 95% CI 0.83-2.42, p=0.201), but a short illness duration decreased them (OR 0.14, 95% CI 0.08-0.27, p<0.001). Presence of vomiting and presence of wheeze both increased prescribing odds (OR_{vomit} 2.17, 95% CI 1.32-3.57, p=0.002; OR_{wheeze} 8.98, 95% CI 4.99-16.15, p<0.001). When prescribing was treated as a 3-category ordinal variable (0=no prescription, 1=delayed prescription, 2=immediate prescription), these findings did not change (Appendix 3).

SfU scores were unrelated to risk assessments (OR 1.00, 95% CI 0.98-1.02, p=0.935; proportional odds assumption met with p_{SfU}=0.406) and prescribing decisions (OR 1.00, 95% CI 0.96-1.03, p=0.875).

DISCUSSION

We compared GPs' risk assessments and antimicrobial prescribing decisions to a normative model (the STARWAVe CPR), in the context of clinical vignettes that varied the features (age, illness duration, vomiting, wheeze) of children presenting with cough. Relative to STARWAVe, GPs frequently underestimated the patient's risk of deterioration, but nonetheless overprescribed: the vast majority of their prescriptions were unnecessary relative to their own risk assessments (78%) and STARWAVe risk assessments (83%).

This is not the first study to observe a disconnect between physicians' risk assessments and antimicrobial prescribing decisions. In one study, for example, an educational intervention was successful in reducing physicians' overestimations of the

For peer review only

likelihood of a bacterial infection, but unsuccessful in reducing antibiotic prescribing.[25] In another, patient expectations for antibiotics increased physicians' rates of antibiotic prescribing, but did not influence their probability estimates of a bacterial infection.[26] Presently, a dissociation between risk assessments and antibiotic prescribing decisions suggests that the former may not be the sole determinant of the latter. It is also possible that explicit risk ratings (as elicited in this type of study) do not reflect physicians' intuitive assessments of risk.

All four of the manipulated variables influenced physicians' (explicit) risk assessments, which increased when the child was younger (20 months vs. 5 years), when illness duration was longer (6 vs. 3 days) and when vomiting and/or wheeze were present (vs. absent). Comparing the odds ratios for these relationships to the STARWAVe model (Table 3), we note both similarities and discrepancies. Specifically, GPs' interpretations of patient age, vomiting and wheeze were consistent with the STARWAVe model, but their interpretation of illness duration was not: a shorter illness duration reduced – rather than increased – GP estimates of risk.

**Predictor**	**OR_{GPs}**	**$OR_{STARWAVe}$**
Age (<2 years)	1.49 [1.14-1.95]*	3.42 [2.12-5.58]*
Duration (≤3 days)	0.54 [0.42-0.69]*	2.77 [1.77-4.35]*
Vomiting	1.92 [1.57-2.36]*	2.56 [1.54-4.31]*
Wheeze	3.33 [2.66-4.16]*	2.16 [1.28-3.60]*
Temperature		1.99 [1.22-3.25]*
Asthma		3.93 [2.20-7.03]*
Recession		3.82 [2.23-6.62]*

For peer review only - http://bmjopen.bmj.com/site/about/guidelines.xhtml

Table 3. The effect of patient age, illness duration, vomiting and wheeze on risk of hospitalisation, according to present participants (OR_{GPs}) and STARWAVe ($OR_{STARWAVe}$). *$p \leq 0.006$. Square brackets contain 95% CIs.

Like risk assessments, prescribing increased when illness duration was long (inverted OR 7.14) and when vomiting and/or wheeze were present (OR_{vomit} 2.17; OR_{wheeze} 8.98). Patient age had no reliable effect on prescribing (OR 1.42). Again, these findings are not entirely consistent with the STARWAVe model, but they are consistent with previous, non-experimental research. In one interview study, for example, GPs reported that they were more likely to prescribe antibiotics to children with RTIs given prolonged duration of symptoms, abnormal chest signs and (less frequently) vomiting.[13] Various observational studies have likewise identified chest abnormalities [27-31] and vomiting [30] as clinical characteristics that prompt prescribing. In contrast, previous literature concerning the effect of age on prescribing is mixed: two studies found that older (vs. younger) patients were more likely to receive a prescription,[29, 32] but three identified no association between age and prescribing.[28, 30, 31]

Interestingly, the one patient that may have warranted a prescription received one only 21% of the time. This appears low, but in fact only 27% of hospitalised children in the STARWAVe cohort had a discharge diagnosis suggestive of a bacterial infection.[12] Consequently, STARWAVe does not argue (or prove) that all high risk children require immediate antimicrobial treatment; rather, it recommends close monitoring and urgent follow-up with a view to prescribe if needed.[12] Viewed thus, the rate of prescription

For peer review only - http://bmjopen.bmj.com/site/about/guidelines.xhtml

that we observed in high risk cases (21%) seems not low, but well-calibrated to the epidemiological landscape (27%).

Risk assessments and prescribing tendencies bore no association to GPs' self-reported levels of "Stress from Uncertainty". However, Grol and colleagues found that greater willingness to take risks (as measured on their Attitudes to Risk Taking scale) was associated with significantly fewer antibiotics prescriptions for respiratory problems and URTI/common cold.[33] Attitudes toward risk – rather than attitudes toward uncertainty – may thus prove a fruitful avenue for future research.

For peer review only

Limitations and future work

This is the first study to identify discrepancies between the STARWAVe clinical prediction rule and current clinical practice. There are several possible reasons for these discrepancies. Firstly, GPs may be unaware of the STARWAVe rule, which was published only four years ago; if so, then dissemination and training may be needed. Alternatively, GPs may be aware of the rule but fail to deploy it at the point of care; in this case, automated STARWAVe support (e.g., incorporation of STARWAVe metrics into the electronic health record) could increase uptake. Even so, the rule is intended to "…supplement, not supplant, clinical judgment" (p. 908)[12] and thus – thirdly – GPs may choose to override it for sound clinical reasons. To illustrate: the major factor triggering prescriptions in the present study was a long illness duration (6 vs. 3 days). This is inconsistent with STARWAVe, but could form part of GPs' strategy to reduce prescriptions, if the alternative is to prescribe early in the illness (i.e., a "wait-and-see" approach). Nonetheless, a more evidence-based strategy is not to prescribe at all in

For peer review only - http://bmjopen.bmj.com/site/about/guidelines.xhtml

simple RTI, which is likely to last longer than 6 days in any case.[20, 34] Finally, it is also possible that methodological aspects of the present study contributed to the discrepancies observed. For example, the distribution of risk in our vignettes (13% very low, 75% medium, 13% high) was not representative of the patient population (67% very low, 30% medium, 3% high [12]) – an unavoidable consequence of our fractional factorial design. In the “real world”, GPs see many more very low risk cases (67% rather than 13%) and fewer medium and high risk cases (30% rather than 75% medium; 3% rather than 13% high). This may have hurt GPs’ performance by being ecologically invalid (i.e., mismatched to true base rates) and could explain their tendency to underestimate risk in the present study.

A more representative set of vignettes would enhance not only the external validity of the study but also the clinical significance of the findings. Our findings speak mostly to the medium risk group (because we employed mostly medium risk cases) but very low risk cases are twice as common in clinical practice, and indeed account for two-thirds of child RTI presentations in primary care.[12] They are also the focal point of the STARWAVe rule, which aims primarily to rule out prescriptions in very low risk cases. The present study employed only one very low risk case and identified a prescription rate of 11%; further work is needed to assess the stability of this estimate in a larger and more varied set of very low risk cases.

While GPs overprescribed relative to STARWAVe guidelines, the rate of prescription identified here (15% across cases) is lower than that observed in other studies. For example, Hay et al. identified a rate of 37% in their prospective cohort study of children presenting to the GP with cough.[12] Notably, present work included few high risk

For peer review only - http://bmjopen.bmj.com/site/about/guidelines.xhtml

presentations (13%), but high risk presentations were likewise infrequent in the study by Hay et al. (3%).[12] If our finding is reflective of real-world practice, then this reduced rate of prescribing is promising indeed. However, it could also reflect the limitations of our vignettes, which ignored the complex interpersonal (doctor-patient) dynamics that are known to influence prescribing behaviour.[13-15, 26, 35] For example, prescription likelihood is increased by perceived pressure from patients/parents to prescribe;[14, 26, 31, 36, 37] by the desire to maintain good relationships with patients/parents;[13, 38, 39] by fear of medicolegal problems;[13, 15, 39] and by time pressure.[13, 14, 37-39] Importantly, these factors can be incorporated into clinical vignettes, as demonstrated by Sirota and colleagues; these authors found that prescriptions were twice as likely when patient pressure for antibiotics was present (vs. absent) from a clinical vignette.[26] On the one hand, it is a limitation of our vignettes that these interpersonal factors were absent; on the other, our work highlights that antibiotics are overprescribed *even when* these interpersonal factors are absent. It is worrying that so many GPs considered antibiotics to be the most appropriate course of action, not simply the most expedient one. Qualitative research may be useful to understand why GPs prescribed to patients that they deemed to be low or medium risk, in the absence of any interpersonal pressure to do so.

Data for this study were collected in conjunction with another project, which limited the number of STARWAVe variables that we could manipulate. A comprehensive investigation of all seven STARWAVe variables would undoubtedly return new and valuable insights. Future investigations might also treat the continuous STARWAVe

For peer review only - http://bmjopen.bmj.com/site/about/guidelines.xhtml

variables (age and illness duration) as continuous (not binary), to test the generalisability of the trends identified here.

A second consequence of collecting data in conjunction with another project is that the antibiotics vignettes (n = 8) were interspersed among many cancer-related vignettes (n = 48). We cannot exclude the possibility that the cancer vignettes influenced performance on the antibiotics task. For example, the cancer vignettes may have primed a hyper-cautious attitude (cancer being a serious, “can’t-miss” diagnosis) that lowered the threshold for intervention (prescription) in the antibiotics task. Threshold for intervention could also be lowered by response fatigue, which participants may well have experienced in assessing so many vignettes. Cognizant of this, we were careful to present the antibiotics vignettes in a random order. Random-ordering would not preclude the cancer vignettes from influencing antibiotics responding; it simply ensured that any such influence was “spread equally” among the antibiotics vignettes.

Despite these limitations, present work sheds light on the determinants of antibiotic prescribing in child RTI presentations, bringing much-needed experimental evidence to a literature that has to date relied predominantly on self-report [13-15, 32, 37, 39, 40] and observational [27-31] data. It also speaks to the difficulties that may be encountered if STARWAVe is provided as a decision aid to GPs. Firstly, GPs’ classification of risk in this study was largely incompatible with STARWAVe’s; GPs consistently chose lower risk than STARWAVe would suggest. Still, they prescribed more frequently than STARWAVe risk classification would support. Presenting GPs with STARWAVe’s risk classification will likely exacerbate prescribing (since GPs

For peer review only - http://bmjopen.bmj.com/site/about/guidelines.xhtml

overprescribed with their own, lower classifications of risk). Presenting them with a recommendation may also be ineffective, unless the recommendation is accompanied by an explanation. Explaining the recommendation in terms of the variables that increase/decrease a child's risk of hospitalisation may be a way forward, and enable GPs to understand why their own intuitive decision might differ from the recommendation. Identifying the factors that are likely to be misinterpreted by GPs is important when explaining the rationale behind recommendations.

COMPETING INTERESTS

Dr. Nurek, Dr. Delaney and Dr. Kostopoulou report grants from the NIHR Imperial Patient Safety Translational Research Centre, during the conduct of the study.

FUNDING

This work was supported by the National Institute for Health Research (NIHR) Patient Safety Translational Research Centre. The views expressed are those of the authors and not necessarily those of the NIHR or the Department of Health and Social Care. The funders had no role in the study design; in the collection, analysis and interpretation of the data; in the writing of the report; and in the decision to submit the paper for publication.

AUTHOR CONTRIBUTIONS

All authors contributed to the design of the study. MN performed the data collection; MN and OK performed the data analysis. MN drafted the manuscript; OK and BD provided critical revision and approved the final version.

DATA SHARING

The data are publicly available on the Open Science Framework under a CC-By Attribution 4.0 International Licence: https://osf.io/r3ype/.

LICENSE STATEMENT

The Submitting Author has the right to grant and does grant on behalf of all authors of the Work (as defined in the below author licence), an exclusive licence and/or a non-exclusive licence for contributions from authors who are: i) UK Crown employees; ii) where BMJ has agreed a CC-BY licence shall apply, and/or iii) in accordance with the terms applicable for US Federal Government officers or employees acting as part of their official duties; on a worldwide, perpetual, irrevocable, royalty-free basis to BMJ Publishing Group Ltd ("BMJ") its licensees and where the relevant Journal is co-owned by BMJ to the co-owners of the Journal, to publish the Work in BMJ Open and any other BMJ products and to exploit all rights, as set out in our licence.

The Submitting Author accepts and understands that any supply made under these terms is made by BMJ to the Submitting Author unless you are acting as an employee on behalf of your employer or a postgraduate student of an affiliated institution which is paying any applicable article publishing charge ("APC") for Open

Access articles. Where the Submitting Author wishes to make the Work available on an Open Access basis (and intends to pay the relevant APC), the terms of reuse of such Open Access shall be governed by a Creative Commons licence – details of these licences and which Creative Commons licence will apply to this Work are set out in our licence referred to above

For peer review only

REFERENCES

1. Department of Health. *UK Five Year Antimicrobial Resistance Strategy 2013 to 2018;* 2013.
2. The White House. *National Strategy for Combating Antibiotic Resistant Bacteria;* 2014. https://www.whitehouse.gov/sites/default/files/docs/carb_national_strategy.pdf. Accessed August 16, 2016.
3. World Health Organisation. *Antimicrobial Resistance: Global Report on Surveillance;* 2014. http://apps.who.int/iris/bitstream/10665/112642/1/9789241564748_eng.pdf. Accessed August 16, 2016.
4. NHS England. *The NHS Atlas of Variation in Healthcare September 2015: Reducing Unwarranted Variation to Increase Value and Improve Quality;* 2015. http://www.rightcare.nhs.uk/atlas/RC_nhsAtlas3_HIGH_150915.pdf. Accessed August 16, 2016.
5. Gulliford MC, Dregan A, Moore MV, et al. Continued high rates of antibiotic prescribing to adults with respiratory tract infection: survey of 568 UK general practices. *BMJ Open* 2014;4(10):e006245.
6. Spinks A, Glasziou PP, Del Mar CB. Antibiotics for sore throat. *Cochrane Database Syst Rev* 2013(11).
7. Smith SM, Fahey T, Smucny J, et al. Antibiotics for acute bronchitis. *Cochrane Database Syst Rev*, 2014(3).
8. Venekamp RP, Sanders SL, Glasziou PP, et al. Antibiotics for acute otitis media in children. *Cochrane Database Syst Rev* 2015(6).

For peer review only

9. Petersen I, Johnson AM, Islam, A, et al. Protective effect of antibiotics against serious complications of common respiratory tract infections: retrospective cohort study with the UK General Practice Research Database. *BMJ* 2007;335(7627):982.

10. Fahey T, Stocks N, Thomas T. Systematic review of the treatment of upper respiratory tract infection. *Arch Dis Child* 1998;79(3):225-230.

11. McCormick A, Fleming D, Charlton J, Office of Population Censuses and Surveys, Department of Health, Royal College of General Practitioners. *Morbidity Statistics from General Practice. Fourth National Study 1991-1992.* London: H.M.S.O.

12. Hay AD, Redmond NM, Turnbull, S, et al. Development and internal validation of a clinical rule to improve antibiotic use in children presenting to primary care with acute respiratory tract infection and cough: a prognostic cohort study. *Lancet Respir Med* 2016;4(11):902-910.

13. Horwood J, Cabral C, Hay AD, et al. Primary care clinician antibiotic prescribing decisions in consultations for children with RTIs: a qualitative interview study. *Br J Gen Pract* 2016;66(644):e207-e213.

14. Lucas PJ, Cabral C, Hay AD, et al. A systematic review of parent and clinician views and perceptions that influence prescribing decisions in relation to acute childhood infections in primary care. *Scand J Prim Health Care* 2015;33(1):11-20.

15. Cabral C, Lucas PJ, Ingram J, et al. “It's safer to…” parent consulting and clinician antibiotic prescribing decisions for children with respiratory tract infections: an analysis across four qualitative studies. *Soc Sci Med* 2015;136:156-164.

For peer review only - http://bmjopen.bmj.com/site/about/guidelines.xhtml

16. Turnbull SL, Redmond NM, Lucas P, et al. The CHICO (Children's Cough) Trial protocol: a feasibility randomised controlled trial investigating the clinical and cost-effectiveness of a complex intervention to improve the management of children presenting to primary care with acute respiratory tract infection. *BMJ Open* 2015;5(9):e008615.

17. Doshi-Velez, F, Kim B. Towards a rigorous science of interpretable machine learning. arXiv:1702.08608, 2017.

18. Chiang PP-C, Glance D, Walker J, et al. Implementing a QCancer risk tool into general practice consultations: an exploratory study using simulated consultations with Australian general practitioners. *Br J Cancer* 2015;112(s1):S77.

19. Wu CFJ, Hamada MS. Experiments: Planning, Analysis, and Optimization. Hoboken, New Jersey: John Wiley & Sons 2009.

20. Guidance from the National Institute for Health and Care Excellence. Respiratory tract infections – antibiotic prescribing: prescribing of antibiotics for self-limiting respiratory tract infections in adults and children in primary care. http://www.nice.org.uk/guidance/cg69. Accessed May 23, 2019.

21. Gerrity MS, White KP, DeVellis, RF, et al. Physicians' Reactions to Uncertainty: refining the constructs and scales. *Motiv Emot* 1995;19(3):175-191.

22. Williams R. Understanding and interpreting generalized ordered logit models. *J Math Sociol* 2016;40(1):7-20.

23. Williams R. Generalized ordered logit/partial proportional odds models for ordinal dependent variables. *Stata J* 2006;6(1):58-82.

24. StataCorp. Stata multilevel mixed-effects reference manual: release 13. College Station, TX;2013.

For peer review only

25. Poses RM, Cebul RD, Wigton RS. You can lead a horse to water - improving physicians' knowledge of probabilities may not affect their decisions. *Med Decis Making* 1995;15(1):65-75.

26. Sirota M, Round T, Samaranayaka S, et al. Expectations for antibiotics increase their prescribing: causal evidence about localized impact. *Health Psychology* 2017;36(4):402.

27. Fischer T, Fischer S, Kochen, MM, et al. Influence of patient symptoms and physical findings on general practitioners' treatment of respiratory tract infections: a direct observation study. *BMC Fam Pract* 2005;6(1):6.

28. Holmes WF, Macfarlane JT, Macfarlane RM, et al. Symptoms, signs, and prescribing for acute lower respiratory tract illness. *Br J Gen Pract* 2001;51(464):177-181.

29. Macfarlane J, Lewis SA, Macfarlane R, et al. Contemporary use of antibiotics in 1089 adults presenting with acute lower respiratory tract illness in general practice in the UK: implications for developing management guidelines. *Respir Med* 1997;91(7):427-434.

30. Hopstaken RM, Butler CC, Muris JWM, et al. Do clinical findings in lower respiratory tract infection help general practitioners prescribe antibiotics appropriately? An observational cohort study in general practice. *Fam Pract* 2005;23(2):180-187.

31. Jakobsen KA, Melbye H, Kelly MJ, et al. Influence of CRP testing and clinical findings on antibiotic prescribing in adults presenting with acute cough in primary care. *Scand J Prim Health Care* 2010;28(4):229-236.

For peer review only

For peer review only - http://bmjopen.bmj.com/site/about/guidelines.xhtml

32. Brookes-Howell L, Hood K, Cooper L, et al. Clinical influences on antibiotic prescribing decisions for lower respiratory tract infection: a nine country qualitative study of variation in care. *BMJ Open* 2012;2(3):e000795.

33. Grol R, Whitfield M, De Maeseneer J, et al. Attitudes to risk taking in medical decision making among British, Dutch and Belgian general practitioners. *Br J Gen Pract* 1990;40(333):134-136.

34. NHS England. *Southwark and Lambeth Antibiotic Guideline for Primary Care 2019*; October 2019. http://www.southwarkccg.nhs.uk/news-and-publications/publications/Medicines%20optimisation/Antibiotic%20guideline%20final%20October%202019.pdf. Accessed March 2, 2020.

35. Howie JG. Clinical judgement and antibiotic use in general practice. *Br Med J*, 1976;2(6043):1061-1064.

36. Mangione-Smith R, McGlynn EA, Elliott MN, et al. The relationship between perceived parental expectations and pediatrician antimicrobial prescribing behavior. *Pediatrics* 1999;103(4):711-718.

37. Dempsey PP, Businger AC, Whaley LE, et al. Primary care clinicians' perceptions about antibiotic prescribing for acute bronchitis: a qualitative study. *BMC Fam Pract* 2014;15(1).

38. Petursson P. GPs' reasons for "non-pharmacological" prescribing of antibiotics: a phenomenological study. *Scand J Prim Health Care* 2005;23(2):120-125.

39. Butler CC, Rollnick S, Pill R, et al. Understanding the culture of prescribing: qualitative study of general practitioners' and patients' perceptions of antibiotics for sore throats. *BMJ* 1998;317(7159):637-642.

40. Kumar S, Little P, Britten N. Why do general practitioners prescribe antibiotics for sore throat? Grounded theory interview study. *BMJ* 2003;326(7381):138.

For peer review only

APPENDIX 1

Characteristics of vignettes

Vignette identifier	Vignette Text	STARWAVe risk assessment	STARWAVe recommendation
1	A 5-year-old child is brought by their parent with a 6-day history of cough. They have no history of asthma or vomiting. On examination, they are pyrexial, and have neither wheeze nor intercostal recession.	Very-low (1 risk factor)	No immediate prescription
2	A 20-month-old child is brought by their parent with a 3-day history of cough. They have no history of asthma or vomiting. On examination, they are pyrexial, and have neither wheeze nor intercostal recession.	Normal (3 risk factors)	No immediate prescription
3	A 20-month-old child is brought by their parent with a 6-day history of cough. They have no history of asthma and have vomited twice in the last 24 hours. On examination, they are pyrexial, and have neither wheeze nor intercostal recession.	Normal (3 risk factors)	No immediate prescription
4	A 5-year-old child is brought by their parent with a 3-day history of cough. They have no history of asthma and have vomited twice in the last 24 hours. On examination, they are pyrexial, and have neither wheeze nor intercostal recession.	Normal (3 risk factors)	No immediate prescription
5	A 20-month-old child is brought by their parent with a 6-day history of cough. They have no history of asthma or vomiting. On examination, they are pyrexial, and have wheeze on chest auscultation. There is no intercostal recession.	Normal (3 risk factors)	No immediate prescription

For peer review only

6	A 5-year-old child is brought by their parent with a 3-day history of cough. They have no history of asthma or vomiting. On examination, they are pyrexial, and have wheeze on chest auscultation. There is no intercostal recession.	Normal (3 risk factors)	No immediate prescription
7	A 5-year-old child is brought by their parent with a 6-day history of cough. They have no history of asthma and have vomited twice in the last 24 hours. On examination, they are pyrexial, and have wheeze on chest auscultation. There is no intercostal recession.	Normal (3 risk factors)	No immediate prescription
8	A 20-month-old child is brought by their parent with a 3-day history of cough. They have no history of asthma and have vomited twice in the last 24 hours. On examination, they are pyrexial, and have wheeze on chest auscultation. There is no intercostal recession.	High (5 risk factors)	Consider an immediate prescription

Note: STARWAVe risk factors are underlined for salience. They were not underlined when vignettes were presented to GPs.

For peer review only

APPENDIX 2

Stress from Uncertainty (SfU) scale

1. I usually feel anxious when I am not sure of a diagnosis.
2. I find the uncertainty involved in patient care disconcerting.
3. Uncertainty in patient care makes me uneasy.
4. I am quite comfortable with the uncertainty in patient care.*
5. The uncertainty of patient care often troubles me.
6. When I am uncertain of a diagnosis, I imagine all sorts of bad scenarios -- patient dies, patient sues, etc.
7. I fear being held accountable for the limits of my knowledge.
8. I worry about malpractice when I do not know a patient's diagnosis.

Note: items 1-5 measure the construct “Anxiety due to Uncertainty” (Cronbach’s alpha=0.86); items 6-8 measure the construct “Concern About Bad Outcomes” (Cronbach’s alpha=0.73).

*Reverse-scored item.

For peer review only

APPENDIX 3

Delayed prescriptions

Delayed prescriptions were administered 12% of the time (121/1008). The association between delayed prescribing and risk (as classified by GPs and by STARWAVe) is displayed below (yellow cells).

		Risk as classified by GPs			STARWAVe risk			Total
		Very-low	Medium	High	Very-low	Medium ("normal")	High	
**Prescriptions**	None	420	294	17	95	551	85	731
	Delayed	25	92	4	17	89	15	121
	Immediate	14	107	35	14	116	26	156
**Total**		459	493	56	126	756	126	1008

To investigate the effect of the manipulated factors on both delayed and immediate prescribing, we regressed the 3-category prescribing variable (0=no prescription, 1=delayed prescription, 2=immediate prescription) on patient age (0=5 years, 1=20 months), illness duration (0=6 days, 1=3 days), vomiting (0=absent, 1=present) and wheeze (0=absent, 1=present). This ordinal logistic regression analysis was conducted using the Stata user-written program "gologit2".[22, 23] Statistical tests of the proportional odds assumption revealed that two variables met it (p_{age}=0.124 and p_{vomit}=0.522) and two did not ($p_{duration}$=0.034 and p_{wheeze}=0.003). Put differently: the respective effects of age and vomiting were consistent for successive levels of the ordinal dependent variable, while those of duration and wheeze were not. Thus, we constructed a partial proportional odds (PPO) model, where two coefficients were fixed (age and vomiting) and two were allowed to vary (duration and wheeze). A global Wald test confirmed that the proportional odds assumption was not violated in this PPO model (χ^2 (2) 2.63, p=0.268).

For peer review only

Results are tabulated below. The model progresses in two steps: the first step compares "no prescription" (coded 0) to "delayed prescription" and "immediate prescription" (both coded 1); the second compares "no prescription" and "delayed prescription" (both coded 0) to "immediate prescription" (coded 1). Trends were consistent across steps, and consistent with those reported in the main text. Specifically, patient age did not influence the odds of a prescription (p=0.569) and short illness duration decreased them (p<0.001). Presence of vomiting and presence of wheeze both increased prescribing odds (both *ps*<0.001). Two coefficients were allowed to vary across steps (duration and wheeze): in both cases, effects grew stronger from step 1 to step 2.

	STEP 1:<br>no prescription (coded 0) vs.<br>delayed/immediate (coded 1)	STEP 2:<br>no/delayed prescription (coded 0)<br>vs. immediate (coded 1)
Age (<2 years)	0.92 [0.69-1.23]	0.92 [0.69-1.23]
Duration (≤3 days)	0.46 [0.34-0.62]*	0.34 [0.24-0.49]*
Vomiting	1.49 [1.24-1.80]*	1.49 [1.24-1.80]*
Wheeze	2.50 [1.91-3.28]*	3.89 [2.66-5.69]*

*p<0.001. Cells contain odds ratios; square brackets contain 95% CIs. Step 2 of the model (no/delayed prescription vs. immediate) is akin to the model reported in the main text; differences in coefficients may be attributed to different estimation procedures (e.g., the ordinal model estimates all parameters simultaneously).[22]

For peer review only

APPENDIX 4

Risk assessments and prescribing decisions per vignette

Vignette identifier	GP risk assessments			GP prescribing decisions		
	Very-low	Medium	High	None	Delayed	Immediate
1	64% (81/126)	35% (44/126)	1% (1/126)	75% (95/126)	14% (17/126)	11% (14/126)
2	71% (89/126)	29% (37/126)	0% (0/126)	94% (118/126)	5% (6/126)	2% (2/126)
3	42% (52/125)	53% (66/125)	6% (7/125)	74% (93/125)	13% (16/125)	13% (16/125)
4	59% (75/127)	40% (51/127)	1% (1/127)	81% (103/127)	14% (18/127)	5% (6/127)
5	25% (32/127)	62% (79/127)	13% (16/127)	59% (75/127)	11% (14/127)	30% (38/127)
6	53% (66/125)	44% (55/125)	3% (4/125)	77% (96/125)	14% (17/125)	10% (12/125)
7	25% (31/126)	64% (80/126)	12% (15/126)	52% (66/126)	14% (18/126)	33% (42/126)
8	26% (33/126)	64% (81/126)	10% (12/126)	67% (85/126)	12% (15/126)	21% (26/126)

For peer review only
